# *Deqi* Sensations of Transcutaneous Electrical Nerve Stimulation on Auricular Points

**DOI:** 10.1155/2013/371543

**Published:** 2013-07-10

**Authors:** Xiaoling Wang, Jiliang Fang, Qing Zhao, Yangyang Fan, Jun Liu, Yang Hong, Honghong Wang, Yunyao Ma, Chunhua Xu, Shan Shi, Jian Kong, Peijing Rong

**Affiliations:** ^1^Guang'anmen Hospital, China Academy of Chinese Medical Sciences, Beijing 100053, China; ^2^Institute of Acupuncture and Moxibustion, China Academy of Chinese Medical Sciences, Beijing 100700, China; ^3^Department of Psychiatry, Massachusetts General Hospital, Harvard Medical School, Boston, MA 02115, USA

## Abstract

*Deqi* sensation, a psychophysical response characterized by a spectrum of different needling sensations, is essential for Chinese acupuncture clinical efficacy. Previous research works have investigated the component of *Deqi* response upon acupuncture on acupoints on the trunk and limbs. However, the characteristics of *Deqi* sensations of transcutaneous electrical nerve stimulation (TENS) on auricular points are seldom reported. In this study, we investigated the individual components of *Deqi* during TENS on auricular concha area and the superior scapha using quantitative measurements in the healthy subjects and depression patients. The most striking characteristics of *Deqi* sensations upon TENS on auricular points were tingling, numbness, and fullness. The frequencies of pressure, warmness, heaviness, and soreness were relatively lower. The dull pain and coolness are rare. The characteristics of *Deqi* were similar for the TENS on concha and on the superior scapha.

## 1. Introduction 

*Deqi* sensation, a psychophysical response, plays a key role in the clinical efficacy of acupuncture according to traditional Chinese medicine [1–4]. Recent neuroimaging fMRI studies also have demonstrated the neural correlates of *Deqi* sensation during acupuncture stimulation [5–8]. The exact components of *Deqi* sensation upon both manual acupuncture and electroacupuncture (EA) have been widely reported in clinical and experimental trials [3, 9–12]. In a survey with international acupuncture experts in 2006, MacPherson and Asghar reported that aching, dullness, heaviness, numbness, radiation, spreading, and tingling closely associate with acupuncture *Deqi* in patients [11]. In another study with 1095 patients, Park et al. found that subjects experienced distension, soreness, pulling, heaviness, tingling, and numbness during acupuncture procedures [12]. Our previous study on *Deqi* sensation of EA in healthy subjects also has revealed that the overall intensities and prevalence of individual sensations are fullness, numbness, soreness, tingling, heaviness, pressure, dull pain, warmness, and coolness in a decreasing order [10]. Overall, these researches of *Deqi* sensation focus on the acupuncture stimulation of the acupoints locating on trunk and limbs. Transcutaneous electrical nerve stimulation (TENS) has recently rapidly developed in the complementary and alternative medicine and it is popular as a kind of EA in acupuncture clinical practice [13, 14]. However, the characteristics of *Deqi *sensation of TENS on auricular points are seldom reported.

TENS on auricular points has been widely used to treat various disorders, for example, epileptic seizures and depression [15, 16]. However, the *Deqi* sensations of TENS on auricular points remain an ongoing area of research. In the present study, we attempted to characterize the *Deqi* sensations including frequency and intensity in individual sensation during TENS on auricular concha area and superior scapha (outer ear margin midpoint) in both healthy subjects and depression patients. This study was a part of the large project of transcutaneous vagus nerve stimulation (tVNS) at auricular concha area for treating major depression [15]. Based on the acupuncture sensation literatures and traditional Chinese medicine textbooks, the selected nine individual sensations in this study were soreness, fullness, numbness, warmness, heaviness, coolness, tingling, pressure, and dull pain. 

## 2. Materials and Methods

### 2.1. Subjects

In the present study, the *Deqi* data were collected as a part of the project of transcutaneous vagus nerve stimulation (tVNS) at auricular concha area for treating major depression. *Deqi *data were recorded during the brain fMRI imaging, which was to investigate the brain effect of tVNS on the depression patients at Guang An Men Hospital. It should be noted that fMRI data will be demonstrated in a separate paper. The hospital's ethics committee approved the research protocol. This study included 15 healthy subjects (18–30 years old, mean ± SD 24 ± 3, 8 M/7 F) and 16 depression patients (18–59 years old, mean ± SD 36 ± 13, 4 M/12 F). Subjects were screened to exclude drug abuse, history of head trauma with loss of consciousness, and contraindications for exposure to high magnetic field. All experimental procedures were explained to the subjects, and signed informed consent was obtained prior to participation in the study. 

### 2.2. Stimulation Procedure

**Figure 1:**
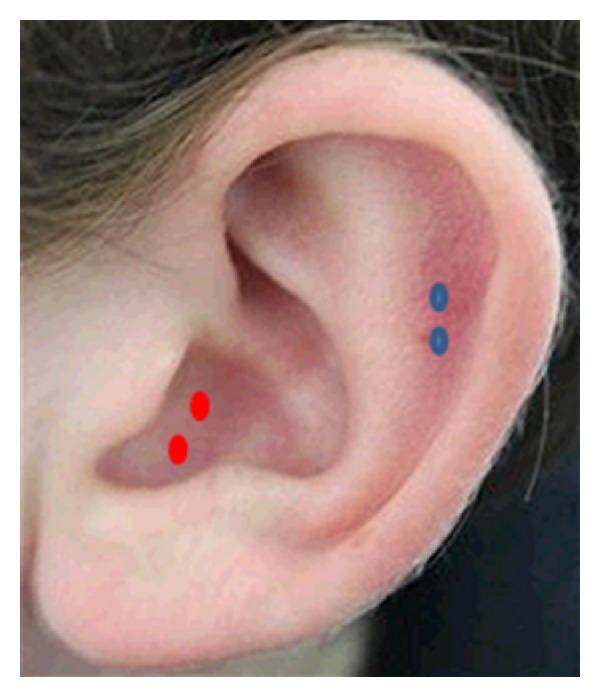
Locations of TENS on the auricular surface. Red spots mean auricular concha area (ACA) and blue spots mean the superior scapha.

The participants were in their supine position in the MRI scanner. The auricular TENS was performed using a stand-alone electrical nerve stimulator, Huatuo (TENS-200, manufactured by Suzhou Medical Appliance Company, Jiangsu, China) connected with nonmagnetic fiber wires to two electrodes. The electrode was attached to the stimulation points with an adhesive tape. The points of TENS included the right auricular concha area (ACA) in both the healthy subjects and depression patients and the right superior scapha (outer ear margin midpoint) only in healthy subjects (Figure 1). The TENS stimulator was placed outside the scanner room. Before each TENS experiment, the stimulator was set at 20 Hz, and the individual threshold of stimulus intensity (mA) was determined, defined by the subjects as a maximum strong sensation that is just not painful and therefore could be well tolerated. Prior to the study, investigators instructed participants about possible *Deqi* sensations and required them to report *Deqi* sensations once they feel it. 

### 2.3. Measurements of *Deqi* Sensations

Epochs of electric current delivery were continuous, lasting for 6 minutes. At the end of the procedure, the participant was questioned by another researcher in the team with a prepared questionnaire if each of the *Deqi *sensations (soreness, fullness, numbness, warmness, heaviness, coolness, tingling, pressure, and dull pain), sharp pain, or any other sensations occurred during the whole process and to rate its intensity on the scale of 0–10 (1–3 mild, 4–6 moderate, 7–9 strong, and 10 unbearable) [9, 10].

### 2.4. Statistical Analysis

Statistical analysis was performed with the SPSS software package 19 (Chicago, Illinois). Two sample paired *t*-tests were performed to compare the best tolerated electric current during TENS between ACA and the superior scapha in the healthy subjects. The paired chi-square tests were performed for comparing the frequencies of individual* Deqi* sensation between two experiments in the healthy subjects. On the other hand, Wilcoxon signed ranks tests were used for the comparison of the intensities in two tests for the healthy group. Independent samples *t*-tests were performed to compare the best tolerated electric current in the ACA between the healthy and patients' groups. The Fisher exact probability tests were used to compare the frequencies of individual *Deqi* sensations during TENS on ACA between the healthy and patients' groups. Mann-Whitney *U* tests were used to compare the intensities of individual *Deqi *sensation in these two groups. 

## 3. Results 

### 3.1. The Best Tolerated Electric Current during Auricular TENS

In the healthy subjects, no significant difference was found in the average amplitude of electric current between TENS on ACA (6.47 ± 0.54 mA) and the superior scapha (6.87 ± 0.41 mA) (*t* = −0.685, *P* = 0.505).

The average amplitude of electric current during TENS on ACA in the depression patients was 5.93 ± 0.36 mA. No significant difference was found between the depression patients and healthy subjects during TENS on ACA (*t* = 0.824, *P* = 0.417). 

### 3.2. Prevalence of *Deqi* Individual Sensations during Auricular TENS in the Healthy Subjects and Depression Patients

**Figure 2:**
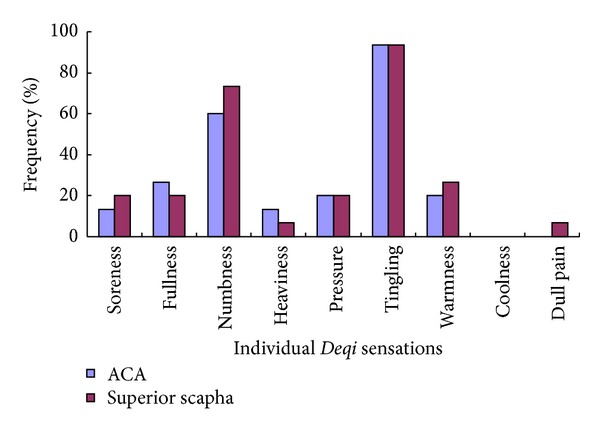
Comparison of the frequency of the individual *Deqi* sensations between TENS on ACA and the superior scapha in the healthy subjects. Tingling, numbness, and fullness were the most common sensations on both ACA and the superior scapha. No significant difference was found between the paired point in the healthy subjects (*P* > 0.05).

The frequency of individual sensation during auricular TENS varies in both healthy subjects and depression patients. However, similar frequencies of tingling, numbness, fullness, pressure, warm, heaviness, soreness, dull pain, and coolness were demonstrated in the healthy subjects for ACA and the superior scapha (*P* > 0.05) (Table 1, Figure 2). 

Sixteen depression patients during auricular TENS on ACA experienced tingling, numbness, fullness, soreness, warm, pressure, and heaviness. None experienced coolness and dull pain (Table 1).

**Figure 3:**
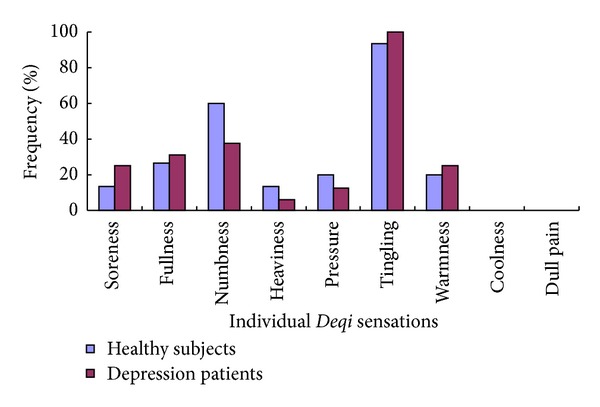
Comparison of the frequency of the individual *Deqi* sensations during TENS on ACA between healthy subjects and depression patients. Tingling, numbness, and fullness were the most common sensations in both healthy subjects and depression patients. No significant difference was found in the individual *Deqi* sensations during auricular TENS on ACA between healthy subjects and depression patients (*P* > 0.05).

No significant difference was found in the nine individual *Deqi* sensations during auricular TENS on ACA between healthy subjects and depression patients (*P* > 0.05) (Figure 3).

### 3.3. Intensity of Individual *Deqi* Sensations during Auricular TENS in the Healthy Subjects and Depression Patients

**Figure 4:**
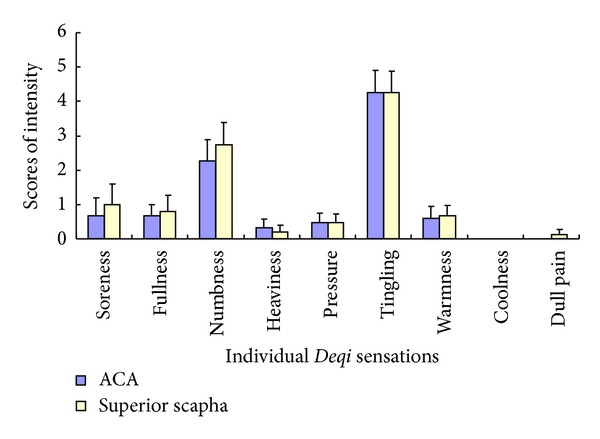
Comparison of the intensity of the individual *Deqi* sensations between TENS on ACA and the superior scapha in the healthy subjects. Tingling and numbness had the greater intensities on both ACA and the superior scapha. No significant difference was found between the paired point in the healthy subjects (*P* > 0.05).

In the healthy subjects, no significant difference was found in the intensity of tingling, numbness, fullness, heaviness, pressure, soreness, dull pain, and warmness between auricular TENS on the two points (*P* > 0.05) (Table 2, Figure 4).

**Figure 5:**
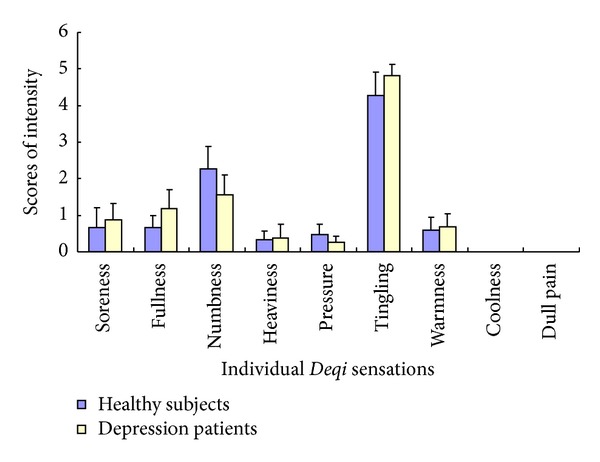
Comparison of the intensity of the individual *Deqi* sensations during TENS on ACA between healthy subjects and depression patients. Tingling and numbness had the greater intensities in both healthy subjects and depression patients. No significant difference was found in the individual *Deqi* sensations during auricular TENS on ACA between healthy subjects and depression patients (*P* > 0.05).

For auricular TENS on ACA, no significant difference was found in the intensity of pressure, numbness, tingling, heaviness, fullness, soreness, and warmness between the healthy subjects and depression patients (*P* > 0.05) (Table 2, Figure 5). 

## 4. Discussion

*Deqi* sensation is a psychophysical response upon acupuncture characterized by a spectrum of different needling sensations which include aching, pressure, soreness, heaviness, fullness, warmth, coolness, numbness, tingling, and dull pain [2, 4, 9–11]. A number of researches have been studying the characterizations of *Deqi* sensations of acupuncture on the acupoints locating at trunk and limbs by penetrating the skin with needles [2, 3, 9–12]. Some studies including ours have been investigating the *Deqi* components using the quantitative measurements [2, 9, 10]. Auricular TENS instead of penetrating has been widely used to treat various disorders like drug addiction, cigarette addiction, mood disorders, obesity, pain, and many other conditions in clinical practice by stimulating points on the ear [15, 16]. However, the characteristics of *Deqi* upon auricular TENS are unclear. In the present study, we investigated the frequency and intensity of *Deqi *individual sensations using the quantitative measurements.

The salient characteristics of *Deqi* upon auricular TENS were tingling, numbness, and fullness. Among these nine individual sensations, the three *Deqi* sensations had both most common prevalence and greater intensities in both healthy subjects and depression patients. The results were consistent with that of EA, which is a form of acupuncture where a small electric current is passed between pairs of acupuncture needles. Leung et al. [17] found that tingling was the predominant *Deqi *sensation deriving from EA. In our previous study using the same quantitative measurement, we found that numbness, fullness, and soreness were the most common sensations during EA on acupoints of ST36, ST28, GB34, CV4, CV12, PC6, and PC7 [10]. TENS is the stimulation with electrodes on the skin instead of insertion of acupuncture needles, and the types of afferent fibers activated by surface electrodes on acupoints correspond to those of EA [18]. EA and TENS are thought to dominantly activate myelinated fibers (A*β* and A*δ*), which is responsible for the numbness and fullness [19, 20]. 

In a study of manual acupuncture, Hui et al. found that the most common individual sensations were soreness, tingling, and numbness [9]. Soreness is less common in the auricular TENS, though it is one of predominant *Deqi* sensations during penetrating the skin with needles. It is well accepted that soreness is correlated with stimulation of small myelinated A*δ* and unmyelinated C fibers instead of A*β* fibers [19, 20].

In the present study, we analyzed the difference of individual *Deqi* sensation between auricular TENS on ACA and the superior scapha in the healthy subjects. No significant difference was found in the prevalence, intensity, and the amplitude of electric current of any individual *Deqi *sensation. It was consistent with the overlapped nerve innervations of the stimulation locations [21]. The ACA is innervated by the auricular branch of vagus nerve and the great auricular nerve. The superior scapha is innervated by the great auricular nerve. The great auricular nerve provides sensory innervations for the skin over both surfaces of the outer ear regions. The posterior branch communicates with the smaller occipital, the auricular branch of the vagus, and the posterior auricular branch of the facial. The overlapping nerve innervations may be responsible for the similar *Deqi *sensations. 

This study was a part of the project of transcutaneous vagus nerve stimulation (tVNS) at ACA for treating major depression [15]. Auricular TENS on ACA has been used to treat depression in clinical practice. In the present study, we also compared the difference of the *Deqi* characteristics of TENS on ACA between the healthy subjects and depression patients. No significant difference was found in the prevalence, intensity, and the amplitude of electric current of any individual *Deqi *sensation. However, the results and the clinical significance need to be further investigated in large cohorts. 

## 5. Limitation

The present results should be explained with caution. The sample size in this study is relatively small. The preliminary results of *Deqi* characterizations on auricular TENS need to be further confirmed by a larger sample size. Moreover, the role of* Deqi* response during auricular TENS in the clinical outcome needs be further investigated.

## 6. Conclusions

The most striking characteristics of *Deqi* sensations upon auricular TENS are tingling, numbness, and fullness. The frequencies of pressure, warmness, heaviness, and soreness are relatively lower. The dull pain and coolness are rare. The characteristics of *Deqi* are similar for the auricular TENS on ACA and on the superior scapha.

## Figures and Tables

**Table 1:** Prevalence of individual *Deqi* sensations during TENS in healthy subjects and depression patients.

	Healthy subject (*n* = 15)		Depression patients (*n* = 16)
	Auricular concha area	Superior scapha	Auricular concha area
Soreness	2	3	4
Fullness	4	3	5
Numbness	9	11	6
Heaviness	2	1	1
Pressure	3	3	2
Tingling	14	14	16
Warmness	3	4	4
Coolness	0	0	0
Dull pain	0	1	0

The paired chi-square tests were performed for comparing the frequencies of individual *Deqi* sensations between the paired TENS points in the healthy subjects (*P* > 0.05). The Fisher exact probability tests were used to compare the frequencies of individual *Deqi* sensations during TENS on auricular concha area between the healthy subjects and depression patients (*P* > 0.05).

**Table 2:** Intensity of individual *Deqi* sensations during EA in healthy subjects and depression patients.

	Healthy subject (*n* = 15)		Depression patients (*n* = 16)
	Auricular concha area	Superior scapha	Auricular concha area
Soreness	0.67 ± 0.54	1 ± 0.47	0.88 ± 0.44
Fullness	0.67 ± 0.32	0.8 ± 0.47	1.19 ± 0.51
Numbness	2.27 ± 0.62	2.73 ± 0.65	1.56 ± 0.55
Heaviness	0.33 ± 0.23	0.2 ± 0.2	0.37 ± 0.37
Pressure	0.47 ± 0.28	0.47 ± 0.29	0.25 ± 0.17
Tingling	4.27 ± 0.64	4.27 ± 0.61	4.81 ± 0.31
Warmness	0.60 ± 0.34	0.60 ± 0.32	0.69 ± 0.36
Coolness	0	0	0
Dull pain	0	0.13 ± 0.13	0

Wilcoxon signed ranks tests were used to compare the intensities of individual *Deqi* sensation between the paired TENS points in the healthy subjects (*P* > 0.05). Mann-Whitney *U* tests were used to compare the intensities of individual *Deqi* sensation during TENS at auricular concha area between the healthy subjects and depression patients (*P* > 0.05).
